# Reduction of seminal plasma concentration can decrease detrimental effects of seminal plasma on chilled ram spermatozoa

**DOI:** 10.1590/1984-3143-AR2020-0211

**Published:** 2021-05-28

**Authors:** Reza Rajabi-Toustani, Mohammad Roostaei-Ali Mehr, Rasool Motamedi-Mojdehi

**Affiliations:** 1 Department of Animal Science, Faculty of Agricultural Sciences, University of Guilan, Rasht, Iran; 2 Department of Clinical Veterinary Science, Obihiro University of Agriculture & Veterinary Medicine, Obihiro, Hokkaido, Japan

**Keywords:** coated spermatozoa, cold-shock, ram spermatozoa, seminal plasma

## Abstract

This study was conducted to investigate the effect of different levels of seminal plasma (SP) and cold-shock on ram spermatozoa during 36 h storage at 5°C. In both ejaculated spermatozoa coated with egg yolk (second ejaculate; coated spermatozoa) and epididymal spermatozoa, samples were treated with 0, 50 and 100% seminal plasma. Different levels of seminal plasma were added on the basis of ram spermatocrit (32%). Then half of aliquots were suddenly put on ice water (cold-shock) and other half were gradually (0.25°C/min) chilled (non- cold shock). Sperm motility, viability and functional membrane integrity were determined in both aliquots at 0, 12, 24 and 36 h storage at 5°C. Under non- cold shock and cold-shock conditions, coated spermatozoa treated with 0% SP showed the highest motility compared to ejaculated spermatozoa (first ejaculate; uncoated spermatozoa) after 12, 24 and 36 h of storage at 5°C (*P*<0.05). Under non- cold shock and cold-shock conditions, viability and functional membrane integrity was higher in the coated spermatozoa treated with 0% SP than in the uncoated spermatozoa during 36 h storage (*P*<0.05). There was no significant difference between coated spermatozoa treated with 0 and 50% SP in the percentage of motility and viability after 24 and 36 h of storage (*P*>0.05). Under non- cold shock and cold-shock conditions, the percentage of motility of epididymal spermatozoa treated with 0% SP was significantly (*P*<0.05) higher than those treated with 100% SP after 36 h of storage at 5°C. In conclusion, removal of seminal plasma and/or reduction (up to 50%) of its concentration can decrease detrimental effects of seminal plasma on chilled ram spermatozoa.

## Introduction

Ram spermatozoa are more sensitive to cold-shock stress than those of other species (Muiño-Blanco et al., 2008). The fertility of liquid stored ram semen following cervical AI rapidly decreases if stored beyond 12–24 h (Maxwell and Salamon, 1993), while acceptable fertility with frozen-thawed ram semen can only be achieved by the use of intrauterine AI (facilitated commercially by laparoscopy; Killen and Caffery, 1982; Maxwell et al., 1984). Therefore, the ability to extend the period of maximum fertility when liquid stored ram semen is used would be of great benefit.

Seminal fluid is a complex medium containing a great variety of molecules, produced by testes, epididymides, sex accessory glands and also cells (apart from spermatozoa) that have many potential effects on both male and female fitness (e.g. sperm capacitation, sperm competition and fertilisation for male, and food, immunostimulation and antibiotic effects for female) (Poiani, 2006). Seminal plasma (SP) can be detrimental to bull and stallion liquid sperm storage (Bergeron et al., 2004; Way and Killian, 2002) or cryopreservation (Martinus et al., 1991; Moore et al., 2005).

Bovine seminal plasma (BSP) protein family binds to sperm membrane choline phospholipids upon ejaculation and stimulates cholesterol and phospholipid efflux from the sperm membrane. In the female reproductive tract, sperm-bound BSP proteins interact with oviductal/follicular fluid components (such as high-density lipoproteins; HDL) and stimulate a second cholesterol efflux, resulting in capacitation (Manjunath and Thérien, 2002). Long exposure of sperm to these proteins or exposure to high concentrations of them could be deleterious to the sperm membrane.

In domestic species, fast cooling from 30 to 0°C causes cell injury in some sperm cells called “cold-shock” that is dependent on the cooling rate and temperature interval (Gilmore et al., 1998; Watson, 2000) and produces an irreversible loss of motility and a disruption of acrosomes and membranes (Kawano et al., 2004).

On the other hand, the discovery that egg yolk has a beneficial effect on fertility of semen (Phillips and Lardy, 1940) led to its widespread use in bull semen extenders. Early researchers reported that egg yolk aids the bull (Lasley and Mayer, 1944) and stallion (Bogart and Mayer, 1950) sperm cell in resisting cold-shock. Also low-density lipoprotein (LDL) would promote the entry of phospholipids and cholesterol into the cell membrane (Bergeron et al., 2004) and build a complex with seminal plasma proteins, making them unavailable to function in the membrane (Bergeron et al., 2004; Manjunath et al., 2002).

A novel method to minimize the damage to bull ejaculated spermatozoa was obtained by coating spermatozoa with the commercial diluents supplemented with egg yolk within 5 min of sperm collection (De Pauw et al., 2003). With this method, the contact between spermatozoa and seminal plasma was limited by collecting bull spermatozoa in a tube that contained diluents supplemented with egg yolk (De Pauw et al., 2003) and the scavenging of BSP proteins by low-density lipoprotein fraction (LDF) contents of egg yolk protects sperm by preventing lipid loss (cholesterol and choline phospholipid) from the sperm membrane (Bergeron et al., 2004).

There is a contradiction in this method that seminal plasma can help sperm cryopreservation and storage or not. Some researchers believe the beneficial effects of seminal plasma (Graham, 1994; Maxwell and Watson, 1996) and others the harmful effects (De Pauw et al., 2003; Roostaei-Ali Mehr et al., 2015; García et al. 2017). This contradictory in regards to removal of the seminal plasma perior to sperm preservation which could improve the motility, viability and plasma membrane integrity of liquid stored ram spermatozoa or not, cause the need of more investigation on it. Therefore, the objective of the present study was to investigate the effect of different levels of seminal plasma and cold-shock on ram spermatozoa during 36 h of storage at 5°C.

## Materials and Methods

### Chemical reagents

The following chemicals were used: tris[hydroxymethyl]aminomethane, citric acid monohydrate, glucose, fructose and sodium citrate dihydrate (AppliChem GmbH, Darmstadt, Germany), nigrosin (Merck, Darmstadt, Germany) and eosin (Panreac, EU, Spain).

### Animals

The study was performed on four Taleshi rams aged between 3 to 5 years. The animals were kept at the University of Guilan, Faculty of Agricultural Sciences, Education Research and Practice Farm, South of Rasht (it is located at 37° 12′ North latitude and 49° 39′ longitude) under uniform housing and lighting conditions. The rams were fed daily with a diet providing 100% of their nutritional needs (NRC, 1985). Animals had free access to salt lick and fresh water. All experimental procedures were performed under the supervision of the Ethics Committee of the University of Guilan.

### Semen collection, dilution and storing

#### Experiment 1

Semen was collected by an artificial vagina from four rams four times at two-day intervals (De Pauw et al., 2003), during the breeding season from autumn to early winter. Within 5 min, the second ejaculates as coated spermatozoa (CS) were collected following the uncoated spermatozoa, using the same method but in a tube containing 1 mL Tris-glucose diluent containing 15% egg yolk. After collection, both samples were transported to the laboratory in an insulated Styrofoam box (33°C) within 45 min after collection.

**Figure 1:**
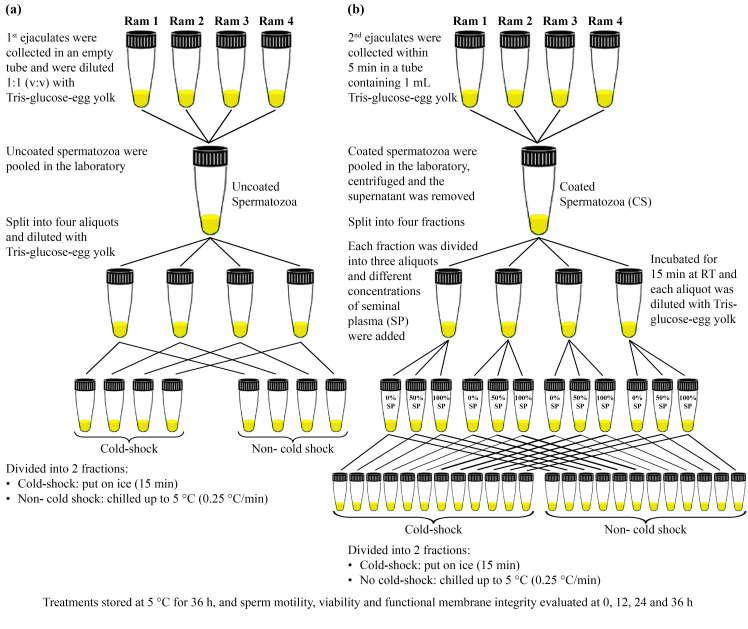
Schematic diagram of the sample preparation procedure in Experiment 1 .

First and second ejaculates were within the criteria of >0.5 mL in volume, sperm with >70% motility and a concentration higher than 2.5×10^9^ sperm/mL were pooled seperately. Uncoated spermatozoa were split into four aliquots and diluted with Tris-glucose diluent containing egg yolk (resulting in final concentrations of egg yolk = 15% and sperm = 600×10^6^ cells/mL). Then, half of aliquots (cold-shock) were suddenly put on ice water for 15 min and other (non- cold shock) was gradually (0.25°C/min) chilled by Test Chamber (EG53AH, KATO, Japan) to 5°C (Rajabi-Toustani et al., 2014). The procedure explained was repeated four times (Figure 1a).

Immediately after primary assessment, CS were pooled and centrifuged for 10 min at 720 ×g at room temperature (RT) to remove the supernatant. CS were split in four fractions and each fraction divided in three aliquots (1200×10^6^ sperm/mL) and 0% SP (seminal plasma) + 100% Tris-glucose diluent (CS_0%SP_), 50% SP + 50% Tris-glucose diluent (CS_50%SP_) and 100% SP + 0% Tris-glucose diluent (CS_100%SP_), added respectively, based on ram spermatocrit (32%) as previously reported (Roostaei-Ali Mehr and Sharafi, 2013) and incubated for 15 min at RT. At last each aliquot was diluted 1:1 (v:v) with Tris-glucose diluent containing 30% egg yolk (resulting in final concentrations of egg yolk = 15% and sperm = 600×10^6^ cells/mL). Then half of aliquots (cold-shock) were suddenly put on ice water for 15 min and another (non- cold shock) was gradually (0.25°C/min) chilled by Test Chamber (EG53AH, KATO, Japan) to 5°C (Rajabi-Toustani et al., 2014). This procedure was repeated four times (r=4; Figure 1b).

#### Experiment 2

**Figure 2:**
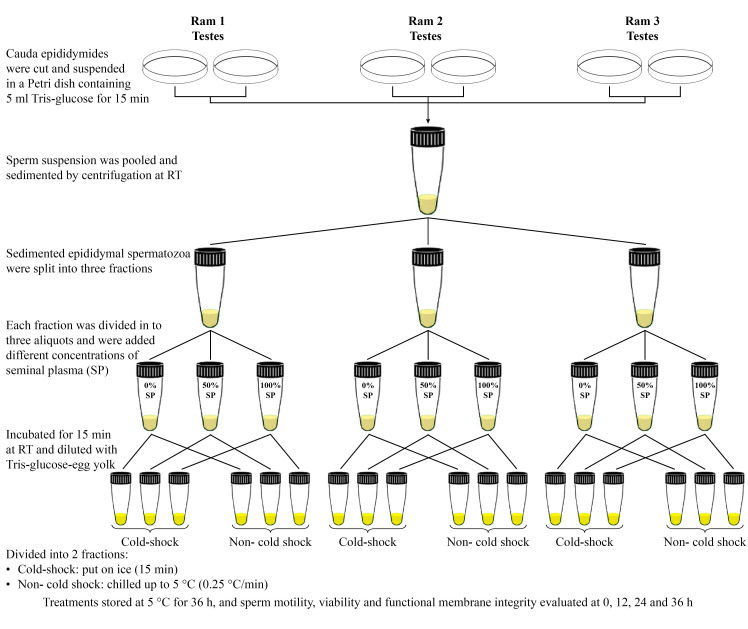
Schematic diagram of the sample preparation procedure in Experiment 2 .

The testes were surgically isolated by open castration under local anesthesia from three rams by a veterinarian. After isolation, the testes were placed in sterile plastic containers, including sterile isotonic normal saline at 35°C, and transported to the laboratory, and mature ram epididymal spermatozoa (EpS) were extracted from the cauda segment of the epididymis within 2 h after surgery. Cauda epididymides were cut with a scalpel into small pieces and were suspended in a Petri dish containing 5 ml Tris-glucose diluent for 15 min. To limit contamination, epididymis samples were carefully dissected free of blood clots and extraneous tissues. Care was taken not to cut blood vessels (Kaabi et al., 2003; Roostaei Ali-Mehr et al., 2012). After that, the sperm suspension was pooled and sedimented by centrifugation at 700 ×g for 10 min at room temperature (RT). This spermatozoa preparation was used for the experiment (Figure 2).

Epididymal spermatozoa were split in three fractions and each fraction divided in three aliquots (1200×10^6^ sperm/mL) and 0% SP + 100% Tris-glucose diluent (EpS_0%SP_), 50% SP + 50% Tris-glucose diluent (EpS_50%SP_) and 100% SP + 0% Tris-glucose diluent (EpS_100%SP_), added respectively, and incubated for 15 min at RT. At last, each aliquot was diluted 1:1 (v:v) with Tris-glucose diluent containing 30% egg yolk (resulting in final concentrations of egg yolk = 15% and sperm = 600×10^6^ cells/ml). Then aliquots were placed under cold-shock and non- cold shock conditions as described in Experiment 1 (Figure 2).

Aliquots of experiments 1 and 2 were kept at 5°C up to 36 h and sperm motility, viability and functional membrane integrity were determined at 0, 12, 24 and 36 h within storage (Figure 2).

### Seminal plasma preparation

For both experiments, ejaculates from four rams were collected, centrifuged (6500 ×g for 15 min at 4°C) and the clear supernatant was recovered. Seminal plasma were pooled, filtered (0.22 µm filter) and stored in 1 mL aliquots at -20°C. Samples were thawed at RT prior to use.

### Sperm assessment

The concentration of spermatozoa was determined by means of a Neubauer haemocytometer.

The percentage of sperm motility was assessed subjectively by phase-contrast microscopy (400×) on a warm stage at 37°C (Evans et al., 1987).

The viability was assessed by means of a one-step eosin-nigrosin staining (Björndahl et al., 2003). Briefly, equal volumes of semen and stain solution (0.67 g eosin Y, 0.9 g sodium chloride and 10 g nigrosin in 100 mL distilled water) were incubated for 30 s at RT (22°C). One drop of mixture was put on a slide, instantly smeared and air dried. A total of 200 sperm were evaluated under light microscope (1000× magnification, oil immersion). Sperm showing partial or complete pink or red colour was considered dead, and sperm showing strict exclusion of the stain was considered to be alive.

The hypo-osmotic swelling test (HOST) was used to evaluate the functional integrity of the sperm membrane. The procedure was described by Jeyendran et al. (1992) and adapted for ram semen by García-Artiga (1994). HOST was performed by incubating 5 μl of semen with 500 μl of a 100 mOsm hypo-osmotic solution (7.35 g sodium citrate dihydrate and 13.51 g fructose in 1 L distilled water) at 37°C for 30 min. One drop of the mixture was placed on a pre-warmed slide, covered with a cover slip and examined under a phase-contrast microscope (400× magnification). The sperm with swollen tails were considered intact. To assess the percentages of intact sperm, a total of 200 sperm were evaluated in at least five different microscopic fields.

### Statistical analysis

All data on motility, viability and functional membrane integrity of sperm were recorded after 0, 12, 24 and 36 h storage at 5°C and analyzed as repeated measures data of GLM procedure of SAS (SAS Institute Inc., 2002) based on completely randomized design. There were four (Uncoated spermatozoa, CS_0%SP_, CS_50%SP_ and CS_100%SP_) and three (EpS_0%SP_, EpS_50%SP_ and EpS_100%SP_) treatments in experiment 1 and 2, respectively. Four times of storage was used as repeated measure. Results are reported as means ± SE. The significance of differences between means was tested at *P*<0.05 by Duncan’s multiple range tests.

## Results

### Experiment 1

**Figure 3:**
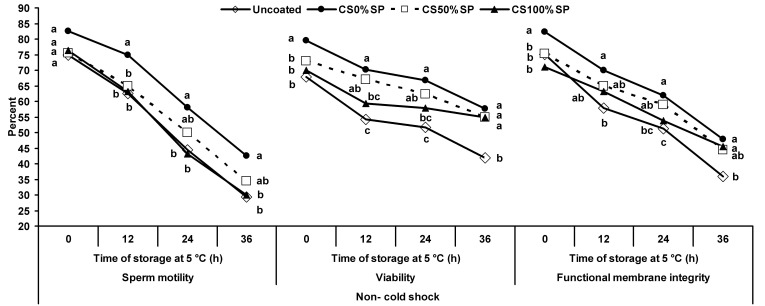
Percentage of motility, viability and functional membrane integrity of ram uncoated (◇) and coated spermatozoa treated with 0 (●), 50 (-□-) or 100% (▲) seminal plasma under non- cold shock conditions during incubation at 5 °C for 36 h. ^a-c^ Different superscripts indicate significant differences among treatments at each time of storage (*P*<0.05).

Under the non- cold shock condition, coated spermatozoa treated with 0% SP showed the higher motility compared to uncoated spermatozoa after 12, 24 and 36 h of storage at 5°C (*P*<0.05; Figure 3). There was no difference between the coated spermatozoa treated with 0 and 50% SP in the percentage of sperm motility (*P*>0.05) at 0, 24 and 36 h of storage (*P*<0.05) respectively. No difference in motility was observed between the coated spermatozoa treated with 50 and 100% SP and the uncoated spermatozoa during 36 h of storage (*P*>0.05). The coated spermatozoa treated with 0 and 50% SP showed the highest viability compared to uncoated spermatozoa after 12, 24 and 36 h of storage (*P*<0.05; Figure 3). There was difference between coated spermatozoa treated with 0 and 50% SP in the percentage of sperm viability at 0 h of storage (*P*<0.05). Uncoated spermatozoa showed the lowest viability compared to other treatments at 36 h of storage (*P*<0.05).

Functional membrane integrity of non- cold shock samples was higher in the coated spermatozoa treated with 0% SP compared to uncoated spermatozoa during 36 h of storage (*P*<0.05; Figure 3).

**Figure 4:**
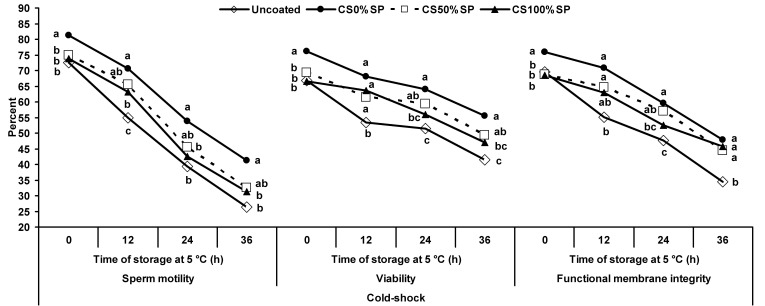
Percentage of motility, viability and functional membrane integrity of ram uncoated (◇) and coated spermatozoa treated with 0 (●), 50 (-□-) or 100% (▲) seminal plasma under cold-shock conditions during incubation at 5 °C for 36 h. ^a-c^ Different superscripts indicate significant differences among treatments at each time of storage (*P*<0.05).

Under cold-shock conditions, coated spermatozoa treated with 0% SP showed the highest motility compared to uncoated spermatozoa during 36 h of storage (*P*<0.05); however, no difference was observed between the coated spermatozoa treated with 0 and 50% SP (*P*>0.05) at 12, 24 and 36 h of storage, respectively (*P*<0.05; Figure 4). There were no differences among the uncoated and the coated spermatozoa treated with 50 and 100% SP in regards to the percentage of sperm motility at 0, 24 and 36 h of storage (*P*>0.05).

When spermatozoa were placed under cold-shock conditions, the percentage of viability of coated spermatozoa treated with 0% SP was higher than uncoated spermatozoa during 36 h of storage at 5°C (*P*<0.05; Figure 4). No difference was observed between coated spermatozoa treated with 100% SP and uncoated spermatozoa after 0, 24 and 36 h of storage (*P*>0.05).

Under cold-shock conditions, coated spermatozoa treated with 0% SP showed the highest functional membrane integrity during the whole period of storage (*P*<0.05; Figure 4). Uncoated spermatozoa showed the lowest functional membrane integrity compared to the other treatments after 36 h of storage (*P*<0.05).

### Experiment 2

**Figure 5:**
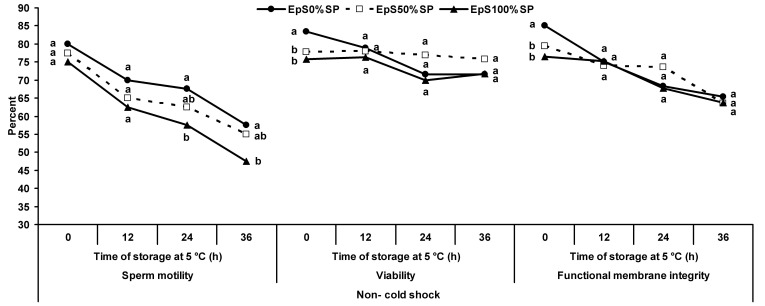
Percentage of motility, viability and functional membrane integrity of ram epididymal spermatozoa treated with 0 (●), 50 (-□-) or 100% (▲) seminal plasma under non- cold shock conditions during incubation at 5 °C for 36 h. ^a-b^ Different superscripts indicate significant differences among treatments at each time of storage (*P*<0.05).

Under the non- cold shock condition, the percentage of motility of epididymal spermatozoa stored with 0% SP was higher than those with 100% SP after 24 and 36 h of storage (*P*<0.05). However, no difference was observed between the epididymal spermatozoa treated with 0 and 50% SP (*P*>0.05; Figure 5).

Under non- cold shock conditions, no difference was observed between the treatments in the percentage of viability and functional membrane integrity (*P*>0.05) except at 0 h of storage, when epididymal spermatozoa treated with 0% SP showed the highest viability and functional membrane integrity (*P*<0.05; Figure 5).

**Figure 6:**
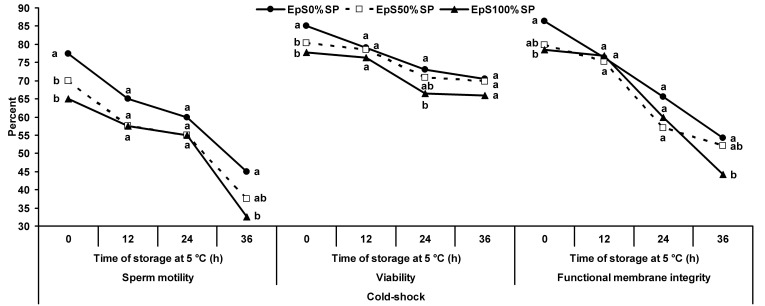
Percentage of motility, viability and functional membrane integrity of ram epididymal spermatozoa treated with 0 (●), 50 (-□-) or 100% (▲) seminal plasma under cold-shock conditions during incubation at 5 °C for 36 h. ^a-b^ Different superscripts indicate significant differences among treatments at each time of storage (*P*<0.05).

Under cold-shock conditions, epididymal spermatozoa treated with 0% SP showed the highest motility and viability at 0 h of storage (*P*<0.05; Figure 6). No difference was observed between the epididymal spermatozoa treated with 0 and 50% SP in the percentage of motility and viability after 36 and 24 h of storage, respectively (*P*>0.05).

When epididymal spermatozoa were placed under cold-shock conditions, the percentage of functional membrane integrity of spermatozoa treated with 0% SP was significantly (*P*<0.05) higher than that with 100% SP after 0 and 36 h of storage (*P*<0.05). No difference was observed between the epididymal spermatozoa treated with 0 and 50% SP (*P*>0.05; Figure 6).

## Discussion

The results of the present study suggested that removal of seminal plasma or reducing the time that the spermatozoa were exposed to seminal plasma increases survival of liquid stored ram semen at 5°C; however, seminal plasma serves as a vehicle for ejaculated sperm, and contains the various proteins and polypeptides that their functions are poorly understood (Manjunath and Thérien, 2002).

Regards of being in non- cold shock or cold-shock conditions, motility, viability and functional membrane integrity was higher in the coated spermatozoa treated with 0% SP than the uncoated spermatozoa during 36 h storage at 5°C. Coated spermatozoa treated with 0% SP had minimum proximity of the spermatozoa and seminal plasma compared to other treatments. Seminal plasma heparin-binding proteins are believed to attach themselves to the sperm surface, especially to the lipids containing a phosphorylcholine group (Calvete et al., 1997), thus allowing heparin-like glycosaminoglycans in the female to trigger sperm capacitation (Nass et al., 1990; Martins et al., 2013). On the other hand, bovine seminal fluid also contains a variety of BSP proteins that bind to phospholipids on the sperm cell membrane, thus modulating the changes that spermatozoa undergo during capacitation (Desnoyers and Manjunath, 1992; Thérien et al., 1995).

The results of the present study agree with findings by De Pauw et al. (2003) that showed that prolonged exposure of spermatozoa to seminal plasma reduces both liquid stored sperm motility and viability and permanently diminishes their fertilizing capacity. Therefore, it is essential that seminal plasma is removed quickly and efficiently. Washing spermatozoa by centrifugal sedimentation and resuspension in fresh medium is generally held as the quickest and most effective method of removing seminal plasma (De Pauw et al., 2003). Our results confirm that detrimental effects of seminal plasma can decrease by reduction of (up to 50%) its concentration.

García et al. (2017) recently shown, sperm washing by centrifugation provided a beneficial effect on ram sperm cryopreservation as seen in sperm viability, membrane integrity and most of the kinetic parameters after thawing. However, the cause for this higher survival could be due to various reasons. One reason may be that the removal of seminal plasma from cryopreservation extenders could improve the protective capacity of the egg yolk against the cold shock as described in different species like rabbit (Ritar and Salamon, 1991), buck (Aboagla and Terada, 2003; Bispo et al., 2011), boar (Bathgate et al., 2006) or horse (Martin et al., 1979), suggesting a negative interaction between seminal plasma and egg yolk, thus affecting motility. Another explanation could be that the technique used to separate seminal plasma through centrifugation may also have eliminated a high percentage of damaged and dead sperm cells (Cebrián et al., 2010).

Spermatozoa recovered from epididymides have suffered the lowest effects of seminal plasma components. As our results showed, the motility of epididymal spermatozoa treated with 0% SP and gradually chilled was higher than 100% SP after 24 and 36 h of storage at 5°C. Epididymal spermatozoa have minimum proximity to seminal plasma and there was no significant difference observed between the treatments in most parameters and different storage times. Other studies have shown that seminal plasma proteins induce the cholesterol efflux of epididymal sperm membrane in bulls (Thérien et al., 1998), boars (Lusignan et al., 2007) and rams (Roostaei-Ali Mehr et al., 2015). BSP protein alone was capable of stimulating cholesterol efflux from bull epididymal sperm in a dose- and time-dependent manner. During the initial 15–30 min of exposure, 7–15% cholesterol was removed, and further exposure of sperm for up to 4 h resulted in about 25% cholesterol removal (Manjunath and Thérien, 2002). Thus long exposure of sperm to these proteins or exposure to large concentrations of them could be deleterious to the sperm membrane and storage. In addition, sperm from species with a low level of cholesterol in their sperm membrane have a decreased tolerance to cold-shock as compared to those with a high level of cholesterol (Darin-Bennett and White, 1977; Drobnis et al., 1993).

In this experiment, the contact time between seminal plasma and spermatozoa was limited by collecting and diluting ram semen directly in Tris-glucose diluent supplemented with egg yolk. Egg yolk is widely used in preservation media, employed for storage of semen from domestic, farm, and zoo animals including endangered species. Phospholipids present in LDL of egg yolk protect sperm by forming a protective film on the sperm surface (Quinn et al., 1980) or by replacing sperm membrane phospholipids that are lost or damaged during the cryopreservation process (Foulkes et al., 1980; Graham and Foote, 1987). Also LDL competes with detrimental seminal plasma cationic peptides (<5 kDa) in binding to the sperm membrane and thus protects the sperm (Vishwanath et al., 1992). On the other hand, milk reduces BSP binding to goat sperm, and such events may explain the protective effect of milk during goat sperm preservation (de Menezes et al., 2016).

While some authors remark the importance of the seminal plasma in the preservation of motility and viability in ram thawed sperm (Graham, 1994; Maxwell and Watson, 1996; van Tilburg et al., 2021) and the importance of seminal plasma heparin-affinity proteins for the success of fertilization (Martins et al., 2013), other researchers describe harmful effects (Schmehl et al., 1986). Detrimental effects of seminal plasma on motility of liquid stored (Lamirande et al., 1984; Dott et al., 1979; Iwamoto et al., 1993) and frozen-thawed (Roostaei-Ali Mehr and Sharafi, 2013) sperm and viability of liquid stored (Dott et al., 1979) and frozen-thawed (Garcia and Graham, 1987; Roostaei-Ali Mehr and Sharafi, 2013) sperm have been reported. These results are similar to our findings that state that the removal of seminal plasma causes beneficial effects on spermatozoa, and results obtained from epididymal spermatozoa are an affirmation of these conclusions. Our results indicated that the coated spermatozoa are better than uncoated spermatozoa and the removal of seminal plasma had a good effect on motility, viability and plasma membrane integrity of ram spermatozoa.

## Conclusion

Seminal plasma act like a double-edged sword being both beneficial and detrimental to sperm depending on different factors such as the concentration and duration of exposure and it requires further research. In conclusion, removal of seminal plasma and/or reduction of (up to 50%) its concentration can decrease the detrimental effects of seminal plasma on chilled ram spermatozoa.
